# Effects of psychoplastogens on blood levels of brain-derived neurotrophic factor (BDNF) in humans: a systematic review and meta-analysis

**DOI:** 10.1038/s41380-024-02830-z

**Published:** 2024-11-29

**Authors:** Abigail E. Calder, Adrian Hase, Gregor Hasler

**Affiliations:** 1 Molecular Psychiatry Lab, Department of Medicine, University of Fribourg, Fribourg, Switzerland; 2 Fribourg Mental Health Network, Chemin du Cardinal-Journet 3, 1752 Villars-sur-Glâne, Switzerland; 3 Lake Lucerne Institute, Vitznau, Switzerland

**Keywords:** Predictive markers, Depression

## Abstract

**Background:**

Peripheral levels of brain-derived neurotrophic factor (BDNF) are often used as a biomarker for the rapid plasticity-promoting effects of ketamine, psychedelics, and other psychoplastogens in humans. However, studies analyzing peripheral BDNF after psychoplastogen exposure show mixed results. In this meta-analysis, we aimed to test whether the rapid upregulation of neuroplasticity seen in preclinical studies is detectable using peripheral BDNF in humans.

**Methods:**

This analysis was pre-registered (PROSPERO ID: CRD42022333096) and funded by the University of Fribourg. We systematically searched PubMed, Web of Science, and PsycINFO to meta-analyze the effects of all available psychoplastogens on peripheral BDNF levels in humans, including ketamine, esketamine, LSD, psilocybin, ayahuasca, DMT, MDMA, scopolamine, and rapastinel. Risk of bias was assessed using Cochrane Risk of Bias Tools. Using meta-regressions and mixed effects models, we additionally analyzed the impact of several potential moderators.

**Results:**

We included 29 studies and found no evidence that psychoplastogens elevate peripheral BDNF levels in humans (SMD = 0.024, *p* = 0.64). This result was not affected by drug, dose, blood fraction, participant age, or psychiatric diagnoses. In general, studies with better-controlled designs and fewer missing values reported smaller effect sizes. Later measurement timepoints showed minimally larger effects on BDNF.

**Conclusion:**

These data suggest that peripheral BDNF levels do not change after psychoplastogen administration in humans. It is possible that peripheral BDNF is not an informative marker of rapid changes in neuroplasticity, or that preclinical findings on psychoplastogens and neuroplasticity may not translate to human subjects. Limitations of this analysis include the reliability and validity of BDNF measurement and low variation in some potential moderators. More precise methods of measuring rapid changes in neuroplasticity, including neuroimaging and stimulation-based methods, are recommended for future studies attempting to translate preclinical findings to humans.

## Introduction

In the late 1990s, the surprising discovery of ketamine’s antidepressant effects spurred a search for the underlying mechanisms [1]. Preclinical research revealed enhanced structural and functional plasticity, particularly in the neocortex, as promising candidates [2]. Relatedly, depressive episodes in both unipolar and bipolar depression are associated with reductions in neuroplasticity at multiple levels of analysis [3, 4]. Research on classic psychedelics and 3,4-Methylenedioxymethamphetamine (MDMA) followed a similar trajectory: the discovery of rapid and sustained therapeutic effects in humans [5] inspired preclinical research which identified promotion of dendritogenesis, synaptogenesis, and increased neurotrophic signaling as potential underlying mechanisms [6–9]. These neuroplastic effects have been most reliably found in the cortex, and specific neural circuits involving the prefrontal cortex appear particularly important [10]. Other regions, including the hippocampus, may also be affected [11]. Psychoplastogens are distinct from other classes of plasticity-promoting drugs due to their rapid, sustained effect and their specificity for particular cortical circuits. Other antidepressants, including selective serotonin reuptake inhibitors and tricyclic antidepressants, also promote neuroplasticity but act more slowly and require chronic administration [12]. Additionally, psychoplastogens are distinct from drugs of abuse that rapidly promote neuroplasticity in dopaminergic neurons and the mesolimbic system, including cocaine and methamphetamine [13]. To distinguish their specific pattern of effects, the term *psychoplastogen* was coined to describe small molecules which rapidly (i.e., within 24 h) and sustainably promote neuroplasticity after a single dose, particularly in prefrontal cortical circuits [14–16]. This rapid, circuit-specific effect is thought to be a therapeutic mechanism in psychedelic-assisted therapy [11, 12].

Psychoplastogens, which may or may not have psychoactive effects, include classic psychedelics such as lysergic acid diethylamide (LSD), psilocybin and psilocin, N,N-dimethyltryptamine (DMT), and 5-MeO-DMT, as well as the entactogen MDMA, NMDA receptor ligands such as ketamine and rapastinel, and the muscarinic receptor antagonist scopolamine [8, 14, 17]. Their neuroplasticity-promoting effects have been associated with behavioral effects relevant to depression and PTSD in animal models [18, 19], as well as reopening of critical learning periods [20]. Further preclinical research has supported the theory that psychoplastogens promote metaplasticity, making the brain more sensitive to environmental inputs and thus to synaptic remodeling [20]. They may also shift the cortex into a hyperplastic state, in which dendritogenesis and synaptogenesis are upregulated for a period of several days, producing new dendrites that can survive for even longer [6, 15]. Nearly all psychoplastogens are thought to have antidepressant effects, and remodeling of the extracellular matrix may be a common mechanism of psychoplastogens from different drug classes [20, 21].

Despite promising preclinical data, testing whether psychoplastogens promote neuroplasticity in humans – and whether this underlies their lasting psychological effects – remains challenging. Many studies with psychoplastogens have used peripheral levels of brain-derived neurotrophic factor (BDNF) as a simple and accessible marker of neuroplasticity [22, 23]. BDNF is one of the most widely expressed and well-studied neurotrophins supporting neuroplasticity, and it has important roles in synaptic and dendritic growth, neuronal maturation, and neuroprotection [24, 25]. Both mature BDNF (mBDNF) and its precursor pro-BDNF may be secreted, and they are both important for various neuroplastic processes [25]. BDNF signaling in the brain is an essential facilitator of learning and memory, and it appears to be important for the action of antidepressant drugs [24]. Increases in BDNF expression in the brain have been observed in rodents after treatment with ketamine, LSD, psilocin, MDMA, and other psychoplastogens [6, 7, 19].

Assessing central BDNF levels is too invasive for most human studies, but BDNF is also present in the bloodstream [23]. Platelets store most of the peripheral BDNF secreted from various tissues, including but not limited to the brain, and a small amount of free BDNF is also present in plasma [26]. Rapid changes in circulating BDNF levels, as might be expected after psychoplastogen administration, may be more detectable in plasma than serum because serum BDNF may mostly be inherited from megakaryocytes at the beginning of platelets’ 7–10 day lifespan [27, 28]. Some studies suggest that brain and peripheral BDNF may correlate with each other and be associated with greater brain volume in the prefrontal cortex, hippocampus, and other regions [29]. However, exactly how much peripheral BDNF originates from the brain remains a matter of debate [30]. BDNF is a relatively large, charged molecule which is tightly regulated at the synapse, though a small amount appears to travel across the blood-brain barrier [31, 32]. However, BDNF produced in other tissues also contributes to peripheral BDNF levels, including the heart, muscle, liver, and platelet precursor cells [30]. Additionally, peripheral BDNF levels show high variability and can be impacted by age, sex, diet, platelet activation, time of day, and handling of blood samples, as well as lifestyle factors such as sleep, exercise, smoking, and drinking [33, 34]. There is also substantial inter-assay variation in many commercial immunoassays used to quantify BDNF [35].

Despite uncertainty about how well peripheral BDNF reflects brain BDNF, it is one of the most widely used markers of neuroplasticity in human studies and seems related to several known correlates of neuroplasticity, including psychiatric disease, stress, and exercise. Studies using peripheral BDNF as a marker have found that it is reduced in depression, anxiety, and several other psychiatric disorders [25, 36, 37], as well as after stroke [38] and in neurodegenerative disorders [39, 40]. Peripheral BDNF levels also increase after aerobic exercise, in line with findings that exercise enhances neuroplasticity in the brain [41], and they may be a marker of exposure to psychosocial stress [42]. Furthermore, BDNF levels normalize after remission from psychiatric disease, particularly in depression but also potentially in other psychiatric diseases, such as eating disorders [43–45]. Several meta-analyses pooling various antidepressant treatments, including pharmacological (selective serotonergic reuptake inhibitors, serotonin-noradrenaline reuptake inhibitors, tricyclic antidepressants) and non-pharmacological treatments (repetitive transcranial magnetic stimulation, vagus nerve stimulation, electroconvulsive therapy), have demonstrated a significant increase in peripheral BDNF after treatment [46–48], though one such analysis–and the only one to include a psychoplastogen, ketamine–also found no change [49]. Generally, BDNF is treated as a sufficient marker of neuroplasticity and is widely used in studies investigating depression and other topics for which neuroplasticity is relevant.

Studies investigating the effects of psychoplastogens on peripheral BDNF have shown mixed results, with some studies showing increases in BDNF after treatment and others showing no change [11]. A recent meta-analysis examined the effects of ketamine and esketamine on peripheral BDNF and other blood-based biomarkers in 11 studies of depressed patients [50]. They reported a significant longitudinal change in BDNF levels amongst treatment responders which was absent in non-responders. Preclinical research using healthy animals suggests that neuroplastic effects may not only be visible in patients, however, and that psychoplastogens other than ketamine should also increase BDNF. Another recent meta-analysis investigated changes in peripheral BDNF after classic psychedelic administration in seven independent samples, mostly healthy subjects. Using a reductionist approach [51] and analyzing only the largest effect size reported from each study (personal communication), they reported an overall increase [52].

Despite their strengths and contributions, previous systematic reviews and meta-analyses have had some limitations. Firstly, none have summarized findings across multiple psychoplastogens. Secondly, authors of some previous meta-analyses reported difficulty in retrieving data from several relevant publications, many of which found no change in BDNF. Finally, some important moderators have not yet been considered, including the timing of BDNF measurements, dose of medications, and aspects of study methodology. Given these limitations, the current analysis includes all available psychoplastogens and several important moderators, as well as a comprehensive search strategy to ensure inclusion of null results. We aimed to thoroughly test the hypothesis that the rapid upregulation of neuroplasticity seen after psychoplastogen administration in preclinical studies is detectable using peripheral BDNF in humans.

## Methods

### Pre-registration

This systematic review was pre-registered in the PROSPERO database before beginning the search process (Record ID: CRD42022333096) [53]. It was conducted according to the guidelines of the Preferred Reporting Items for Systematic Reviews and Meta-Analysis (PRISMA) [54].

### Inclusion criteria

We searched for clinical studies in adult human subjects which evaluated changes in peripheral BDNF after administration of a psychoplastogen. We included all psychoplastogens which have been shown to promote neuroplasticity in animal studies, including LSD, psilocybin and psilocin, DMT (including ayahuasca), 5-MeO-DMT, subanesthetic ketamine, MDMA, scopolamine, and rapastinel. Though we were primarily interested in effects within the first 24 h, we included all available timepoints for completeness. To provide specificity, we excluded studies in which the psychoplastogen was systematically combined with another pharmacological or neurophysiological intervention in all treatment arms, for example other antidepressants, electroconvulsive therapy, or transcranial magnetic stimulation. Studies incorporating psychotherapy or psychological support were not excluded, nor were studies in which participants continued pre-existing psychiatric medications. Additionally, we excluded studies in which there was no comparison group (either placebo or baseline). We only included English language studies published in peer-reviewed journals since the year 1982, the year in which BDNF was first identified [55].

### Search strategy

The initial search was conducted on May 25th, 2022 and updated on June 13th, 2023. We searched the electronic databases PubMed, Web of Science, and PsycINFO. Where possible, we tailored the search to each database to filter by our inclusion criteria. For all three databases, we used the filters for English language and peer-reviewed journals and restricted the publication date to after 1982. In PubMed, we additionally used the filters for clinical studies and human subjects.

We used broad inclusion criteria for our initial search because we had observed that some clinical studies of psychoplastogens which measured BDNF did not appear in search results when filtering by the keyword “BDNF”, “neurotrophic”, or other terms related to neuroplasticity, even when search filters were set to “all fields”. This appeared to occur most often for studies which did not report a significant result for BDNF, particularly when BDNF was not a main outcome and thus did not feature in the abstract. We therefore decided to first identify all clinical studies in human subjects in which any psychoplastogen had been administered, then manually search the full text for keywords related to BDNF. First, we searched databases for articles containing the following keywords in the title or abstract: “psychedelic” OR “psilocybin” OR “psilocin” OR “LSD” OR “lysergic acid diethylamide” OR “DMT” OR “N,N dimethyltryptamine” OR “5-MeO-DMT” OR “5-methoxy-N,N dimethyltryptamine” OR “ketamine” OR “MDMA” OR “3,4-methylenedioxymethamphetamine” OR “ayahuasca” OR “rapastinel” OR “scopolamine” OR “psychoplastogen”. We retrieved the title and abstract of all relevant articles.

Second, two reviewers (AEC and AH) filtered the results by title and abstract using a combination of automatic and manual searching. The goal was to identify studies in which a psychoplastogen was administered to human subjects which could then be manually searched for keywords related to BDNF. Automatic filters were applied to remove articles with common keywords in the title demonstrating that the study could not be included. These included keywords only seen in titles of animal studies (e.g. “rat”, “mouse“, “horse“), keywords related to pediatrics (“pediatric“, “children”), keywords related to the use of concomitant interventions (e.g. “propofol”, “fentanyl”, “opioid”), and keywords related to surgical interventions (e.g. “anesthetic”, “anesthesia”, “surgical”). The remaining articles were filtered manually using both the title and the abstract. Discrepancies were resolved by discussion.

Finally, the same two reviewers retrieved and electronically searched the full text of remaining articles for keywords related to markers of BDNF (namely, “neurotrophic” and “BDNF”). We included all articles which assessed BDNF in adult humans after treatment with a psychoplastogen and included a control group (placebo and/or baseline BDNF levels). Discrepancies were resolved by discussion.

### Risk of bias assessment

We performed a risk of bias assessment using the Cochrane Risk of Bias Tools for parallel group and crossover designs [56], as well as the ROBINS-I tool for open-label trials [57]. The assessment was performed by two authors (AEC and AH) separately, and discrepancies were resolved by discussion.

### Data extraction

The main outcome of this analysis was change in peripheral BDNF after treatment with a psychoplastogen. From each paper, we recorded the sample size and the mean and standard deviation for BDNF values. Many papers reported multiple effect sizes for different drugs, doses, and timepoints. We chose an integrative meta-analytic approach in order to analyze the impact of potential moderators, because BDNF values are known to be variable and meta-analyses can be strongly affected by choices to only include particular effect sizes [51]. We thus extracted data for all available psychoplastogens, doses, and timepoints. When the required BDNF data was not present in a publication, we requested it from the authors. If data could not be obtained this way, we extracted it from plots using WebPlotDigitizer (Version 4.6). If the required data could still not be obtained, the affected study was excluded from the meta-analysis.

Additionally, we extracted data concerning participant characteristics (age, gender, diagnoses), the specific psychoplastogen(s) and doses used, blood fraction used (i.e., serum or plasma), study design, percentage of missing data, and the timing of each BDNF measurement, including whether it was taken after a single dose or after repeated doses, which was common in open-label ketamine studies. Some studies involving ketamine also divided the sample into responders and non-responders. When possible, we analyzed a single effect size for the whole sample, otherwise we included all effect sizes available in the mixed effects model. We chose this route firstly because an analysis of BDNF in ketamine responders and non-responders has recently been done [50], and secondly because preclinical studies report an overall increase in BDNF without dividing samples. Additionally, studies varied in their choices of controls, with some using only a baseline BDNF measurement, some a comparison to placebo, and some both. We included the strongest control available, with placebo control relativized to baseline being the strongest and placebo control alone the second strongest. Baseline only was considered the weakest control because BDNF levels tend to decrease throughout the day [33] and follow-up measurements were often taken later in the day than baseline.

### Statistical analyses

Using Hedges’ *g* as the standardized mean difference (SMD), we configured a mixed effects meta-analysis with random effects for each individual study and effect size. Test statistics and confidence intervals were obtained with cluster robust variance estimation. We first performed a meta-analysis for all psychoplastogens together, then individually for each drug that was represented by at least three independent studies (namely ketamine, LSD, and psilocybin). Additionally, we analyzed all classic psychedelics together in an attempt to replicate results from a previous meta-analysis [52]. When sufficient data were available, we also performed subgroup analyses and meta-regressions to analyze the impact of potential moderators. Subgroup analyses were conducted for plasma and serum because rapid changes in BDNF might appear sooner in plasma. Further subgroup analyses were conducted for healthy subjects and depressed patients, including those with bipolar depression, because the reduced neuroplasticity observed in depressive episodes may affect the response to psychoplastogens. Meta-regressions were used to analyze the impact of the following variables: (1) timing of post-treatment BDNF measurements in hours; (2) average participant age; (3) type of control group, i.e. baseline only, placebo only, or both; (4) overall study design, i.e. parallel group design, crossover, or non-randomized (i.e., open-label); (5) whether BDNF was measured after a single dose or after repeated dosing; (6) immunoassay specificity to mBDNF, pro-BDNF, or both; (7) overall risk of bias rating; (8) percentage of missing BDNF values; and (9) dose, for the analyses involving single drugs and routes of administration. Publication bias was assessed using Egger’s tests combined with visual inspection of funnel plots. All statistical analyses were done in R (Version 4.2.3) using the metafor package (Version 4.4.0) [58]. Forest plots were generated using the meta package [59].

## Results

### Included studies

**Fig. 1:**
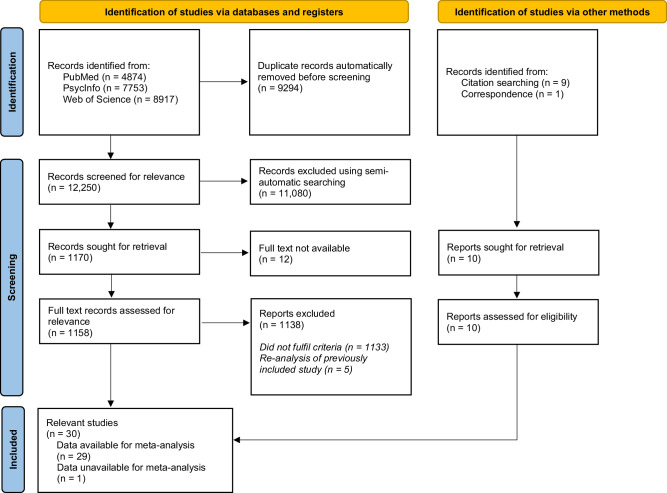
PRISMA diagram showing the search process.

The initial search identified 13,094 records after removal of duplicates (Fig. 1). Of these, 1170 were identified as clinical studies in which psychoplastogens were administered to adult human subjects. We identified 29 studies which measured BDNF levels after psychoplastogen administration, compared these to a control condition, and had available data for meta-analysis (Table 1). A total of 104 effect sizes from these 29 studies were included in the meta-analysis. Studies contributing multiple effect sizes (N = 22) had effect sizes for different timepoints, doses, and/or drugs.

**Table 1:** Studies included in the meta-analysis. N is the sample size included in this analysis.

Study	N	Drug	Dose	Timepoints	Sample	Blood fraction	BDNF change
Allen et al. [102]	16	ketamine	0.5 mg/kg	2, 24 h; 7 d	TRD	serum	No change except at 7 d in responders
Becker et al. [103]	23	psilocybin	25 mg	4, 7 h	healthy	plasma	Increase
Becker et al. [62]	24	LSD	100 µg	6, 9, 12 h	healthy	plasma	Increase
Caliman-Fontes et al. [104]	27	esketamine	0.25 mg/kg	24 h, 7 d	TRD	serum	No change
		ketamine	0.5 mg/kg				
de Almeida et al. [105]	69	ayahuasca	1 mL/kg	48 h	healthy, TRD	serum	Increase
Duncan et al. [106]	29	ketamine	0.5 mg/kg	4 h	MDD	plasma	Increase
Glue et al. [107]	6	ketamine	60, 120, 240 mg p.o.	24 h	healthy	serum	No change
Glue et al. [108]	12	ketamine	0.25, 0.5, 1 mg/kg	0.25, 0.5, 1, 2 h	anxiety	serum	No change
Glue et al. [109]	7	ketamine	60 mg p.o.	2-96 h	TRD, anxiety	serum	No change
Grunebaum et al. [110]	46	ketamine	0.5 mg/kg	4 h	MDD	plasma	No change
Grunebaum et al. [111]	9	ketamine	0.5 mg/kg	4 h	BPD	plasma	No change
Haile et al. [112]	15	ketamine	0.5 mg/kg	4 h	TRD	plasma	Increase in responders only
Holze et al. [60]	27	LSD	100 µg	3, 5 h	healthy	plasma	No change
		MDMA	100 mg				
Holze et al. [113]	16	LSD	25, 50, 100, 200 µg	6, 12, 24 h	healthy	plasma	Increase after 200 µg LSD only
Holze et al. [114]	28	LSD	100, 200 µg	4, 6, 12 h	healthy	plasma	No change
		psilocybin	15, 30 mg				
Hutten et al. [63]	10	LSD	5, 10, 20 µg	2, 4, 6 h	healthy	plasma	Increase after 5 and 20 µg LSD only
Kang & Vasquez [115]	10	ketamine	0.5 mg/kg	7 d	TRD	serum	No change
Ley et al. [116]	32	LSD	100 µg	3, 6, 12 h	healthy	plasma	No change
		psilocybin	20 mg				
		mescaline	300, 500 mg				
Linkovski et al. [117]	7	ketamine	0.5 mg/kg	0.67, 2, 4 h	OCD	serum	Decrease in rapastinel group
		rapastinel	10 mg/kg	0.67, 2, 4 h			
Machado-Vieira et al. [118]	23	ketamine	0.5 mg/kg	4 h	healthy	plasma	No change
Medeiros et al. [119]	38	ketamine	0.5 mg/kg	4, 24, 72 h	MDD	plasma	No change
Park et al. [120]	23	scopolamine	4 µg/kg	2.5 h	MDD	plasma	No change
Rocha et al. [121]	15	ayahuasca	1 mL/kg	2, 4 h	healthy	plasma	No change
Rybakowski et al. [122]	25	ketamine	0.5 mg/kg	7, 14 d	BPD	serum	Decrease in non-responders only
Straumann et al. [61]	24	LSD	100 µg	3, 6, 9, 12, 24 h	healthy	serum	No change
		MDMA	100 mg				
Vogt et al. [123]	27	DMT		1.5, 2.5 h	healthy	plasma	No change
Wang et al. [124]	127	ketamine	0.5 mg/kg	13, 26 d	BPD, MDD	serum	Increase in responders at day 14 only
Woelfer et al. [125]	36	ketamine	0.5 mg/kg	2, 24 h	healthy	plasma, serum	Increase
Zheng et al. [126]	75	ketamine	0.5 mg/kg	13, 26 d	MDD	plasma	Increase
**Total**	**826**						

Timepoints refers to the timing of blood draws used for BDNF analysis. BDNF change refers to the change compared to the control condition used for this analysis.

*TRD* treatment-resistant depression, *MDD* major depressive disorder, *BPD* bipolar depression, *OCD* obsessive compulsive disorder.

The majority of these studies administered ketamine (N = 17), though some also used LSD (N = 7), psilocybin (N = 3), ayahuasca (N = 2), MDMA (N = 2), and other psychoplastogens (N = 1 each for esketamine, DMT, mescaline, rapastinel, and scopolamine). Most studies analyzed BDNF levels after a single dose of a psychoplastogen, but six studies analyzed BDNF after repeated infusions (range: 3–7) of ketamine (N = 5) or rapastinel (N = 1). Fourteen studies involved healthy subjects and 16 involved patient groups, including patients with depression (N = 12), bipolar depression (N = 3), anxiety disorders (N = 2), and OCD (N = 1). Most studies measured BDNF in the first 48 h after drug administration (N = 25), and a few ketamine studies measured it between 7 and 26 days later (N = 6). We could find information on the specificity of immunoassays for mBDNF and pro-BDNF for 23 studies, of which 13 assessed total BDNF and 10 (all from the same research group) used an assay specific to mBDNF (Table S1).

### Risk of bias assessment

Results of the risk of bias assessment are shown in Supplementary Fig. S1. For the 12 randomized crossover trials, one was rated as high risk of bias, and the rest were rated as low risk. For the nine randomized parallel trials, four were rated as low risk, three as having some concerns, and two as high risk. The most problematic domains involved missing outcome data and potential for selection of the reported result. For non-randomized trials, all eight studies received a high/serious risk of bias rating, largely due to the potential for baseline confounding and potential for bias in the selection of the reported result.

### Effects of psychoplastogens on BDNF

**Fig. 2:**
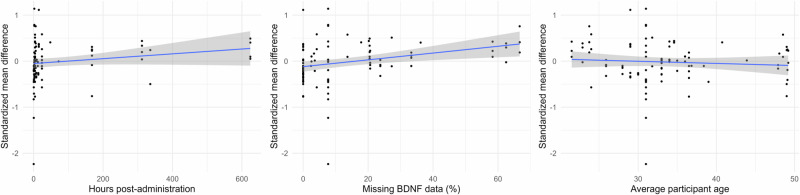
Relationship between effect sizes and the continuous variables of timing, percentage of missing values, and average participant age for studies administering any psychoplastogen. Shading shows 95% confidence intervals. The effect of hours post-administration was significant but minimal (SMD = 0.0005, p = 0.038). Studies with a higher percentage of missing data reported significantly greater effect sizes (SMD = 0.77, p = 0.028).

Results from all analyses, including subgroup analyses and metaregressions, are summarized in Table 2. The combined effect size across all psychoplastogens was not significant (SMD = 0.024, *p* = 0.64). There was a relatively low level of heterogeneity (I^2^ = 22.64%) and no evidence of publication bias (*p* = 0.15, Supplementary Fig. S2). Subgroup analyses found no significant effects for plasma or serum alone, as well as no effect for healthy subjects or depressed patients alone. Because of the possibility that short-term changes in BDNF (<7 days post-treatment) may be better visible in plasma and long-term changes (≥7 days post-treatment) may be better visible in serum, we repeated the analyses using only data on short-term changes in plasma and long-term changes in serum; this did not change any of the results. Meta-regressions found no significant effect of age, study design, BDNF immunoassay sensitivity, or overall risk of bias on change in BDNF. Later BDNF measurements were associated with minimally larger effect sizes (SMD = 0.0005, *p* = 0.038, Fig. 2). Studies comparing change from baseline BDNF between a treatment and placebo group reported significantly lower effect sizes than studies using only a baseline BDNF measurement as a control group (SMD = -0.31, *p* = 0.031). Additionally, studies with a higher percentage of missing data reported greater effect sizes (SMD = 0.77, *p* = 0.029), and studies with a high overall risk of bias rating showed significantly greater effect sizes than studies with a low rating (SMD = 0.26, *p* = 0.017).

**Table 2:** Summary of all results from subgroup analyses and meta-regression models analyzing the effects of psychoplastogens on peripheral BDNF.

Subgroup analyses					
	Overall	Healthy	Depression	Plasma	Serum
All psychoplastogens	0.02 [–0.08 to 0.13]	–0.01 [–0.17 to 0.14]	0.10 [–0.06 to 0.26]	0.05 [–0.09 to 0.19]	–0.04 [–0.21 to 0.13]
Ketamine	0.03 [–0.11 to 0.17]	–0.08 [–0.25 to 0.08]	0.10 [–0.07 to 0.27]	0.09 [–0.17 to 0.34]	–0.05 [–0.26 to 0.17]
Classic psychedelics	0.05 [–0.16 to 0.26]	–	–	0.04 [–0.20 to 0.28]	0.09 [–4.15 to 4.33]

Meta-regressions						
	Dose	Timing (h)	Age	Missing values	mBDNF	Multiple infusions
All psychoplastogens	–	0.0005* [0–0.001]	–0.0008 [–0.01 to 0.01]	0.77* [0.13–1.42]	0.007 [–0.26 to 0.27]	–
Ketamine	–	0.0007* [0.0002–0.001]	–0.003 [–0.02 to 0.01]	0.76 [–0.18 to 1.70]	0.10 [–0.14 to 0.34]	0.26 [–0.05 to 0.56]
Classic psychedelics	–	0.01 [–0.01 to 0.03]	0.005 [–0.08 to 0.09]	0.68 [–2.01 to 3.37]	–0.32 [–1.43 to 0.79]	–
LSD	0.002 [–0.01 to 0.01]	0.02 [–0.04 to 0.09]	–0.03 [–0.09 to 0.03]	0.59 [–1.26 to 2.44]	–	–
Psilocybin	0.006 [–0.37 to 0.38]	–0.06 [–0.33 to 0.22]	0.09 [–0.32 to 0.51]	7.06 [–3.43 to 17.56]	–	–
	**Design**		**Control group**		**Risk of bias**	
	**Crossover RCT**	**Parallel RCT**	**Placebo**	**Baseline + placebo**	**Some concerns**	**High**
All psychoplastogens	–0.18 [–0.44 to 0.08]	–0.13 [–0.38 to 0.11]	–0.006 [–0.27 to 0.25]	-0.31* [–0.55 to –0.06]	–0.005 [–0.23 to 0.22]	0.26* [0.05–0.46]
Ketamine	–0.46 [–1.12 to 0.20]	–0.26* [–0.48 to –0.04]	–0.19 [–1.53 to 1.15]	–	0.04 [–0.22 to 0.31]	0.32* [0.02–0.62]
Classic psychedelics	–0.32 [–1.43 to 0.79]	–	0.005 [–1.07 to 1.08]	–0.40 [–1.19 to 0.39]	–	0.35 [–0.04 to 0.74]
LSD	–	–	0.13 [–0.45 to 0.71]	–0.32 [–0.91 to 0.28]	–	0.34 [–0.02–0.71]

Missing fields indicate insufficient data available for analysis. Data are shown as standardized mean difference with 95% confidence intervals. In the moderator analyses, contrasts were as follows: mBDNF vs. total BDNF, multiple vs. single infusions, crossover or parallel RCT vs. non-randomized open-label trials, placebo control groups vs. baseline only control, and some concerns or high risk of bias vs. low risk of bias. *p < 0.05.

**Fig. 3:**
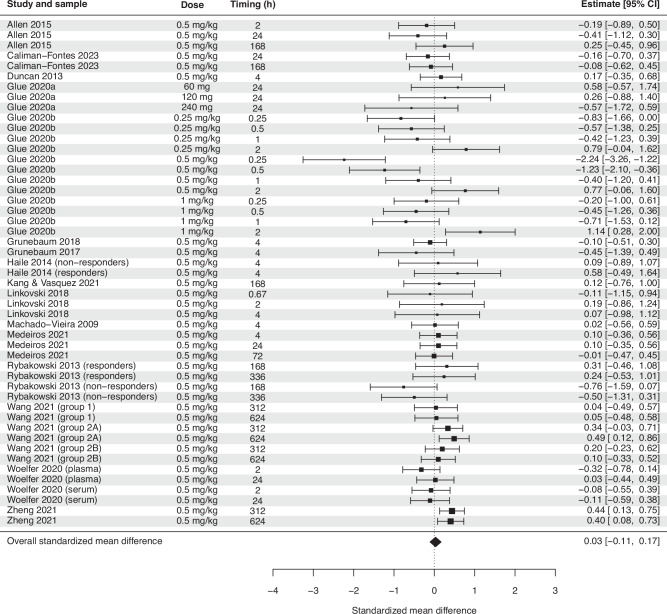
Forest plot showing overall effect size estimates of the change in BDNF after administration of ketamine. There was no significant effect of ketamine on BDNF.

For ketamine, the overall effect size was not significant (SMD = 0.03, *p* = 0.65, Fig. 3). Subgroup analyses found no effect for plasma or serum, as well as no effect for healthy subjects or depressed patients (Table 2). There was no significant effect of age, missing values, control group, immunoassay sensitivity, or repeated infusions. Randomized trials using a parallel design reported significantly smaller effect sizes than non-randomized open-label trials (SMD = –0.26, *p* = 0.03), and trials with a high risk of bias rating reported significantly higher effect sizes than those with a low rating (SMD = 0.32, *p* = 0.04). Later BDNF measurements showed minimally larger effect sizes (SMD = 0.0007, *p* = 0.02, Supplementary Fig. S3).

**Fig. 4:**
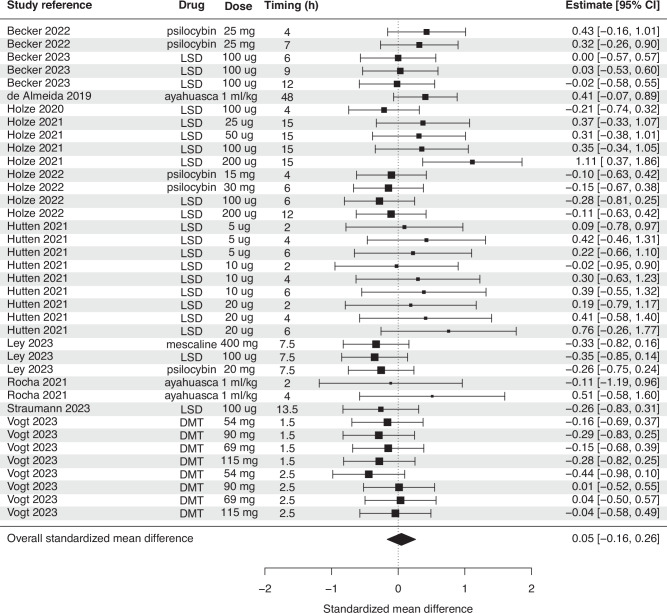
Forest plot showing overall effect size estimates of the change in BDNF after administration of classic psychedelics. There was no significant effect of psychedelics on BDNF.

Classic psychedelics, when analyzed together, also had no significant effect on BDNF (SMD = 0.048, *p* = 0.62, Fig. 4). There was also no effect for either plasma or serum alone (Table 2). Meta-regressions showed no significant effect of timing, age, study design, missing values, control condition, or immunoassay specificity. When analyzing individual psychedelics, we also found no significant effect for LSD (SMD = 0.01, *p* = 0.93, Supplementary Fig. S4) or psilocybin (SMD = 0.007, *p* = 0.97, Supplementary Fig. S5). For LSD, meta-regressions showed no significant effect of dose, timing, control group, age, or missing values (Supplementary Fig. S6), although there was a trend toward studies with higher risk of bias having greater effect sizes than studies with low risk (SMD = 0.34, *p* = 0.06).

## Discussion

This meta-analysis found no evidence that a single dose of any psychoplastogen, including ketamine, LSD, psilocybin, and others, rapidly increased peripheral BDNF levels in human subjects. This is the first meta-analysis to consider all available psychoplastogens and timepoints, as well as both patients and healthy people, when investigating effects of psychoplastogens on peripheral BDNF. Furthermore, a major strength of this analysis is the particular care given to searching for null results which might otherwise be under-represented, as well as the use of mixed effects models including all reported effect sizes in published papers and the consideration of moderators.

The impact of psychoplastogens on BDNF did not change based on specific drugs or drug classes, dose, blood fraction, psychiatric diagnoses, or average participant age. We were not able to replicate previous reports suggesting that classic psychedelics elevate peripheral BDNF levels [52]. This may be due to our inclusion of all available means for all drugs, doses, and timepoints in each study, as well as the inclusion of four additional studies of classic psychedelics [60–63] and differences in statistical choices when selecting data for calculating standardized mean differences.

We found a small but significant effect of timing, so that a greater amount of time between the psychoplastogen intervention and blood collection was associated with slightly larger effect sizes. Though the effect was minimal, it would be consistent with a previous meta-analysis suggesting that responders to ketamine treatment for depression showed greater longitudinal increases in peripheral BDNF over time [50]. Preclinical studies suggest that psychoplastogens induce rapid and sustained upregulation in brain BDNF signaling which is maintained via prolonged mTOR activation [16]. Assuming a similar process occurs in humans, it may take time for the increase in neural BDNF to be reflected in the periphery, and peripheral BDNF may thus not reflect the nearly immediate antidepressant effects of ketamine or other psychoplastogens. It is also possible that late increases in peripheral BDNF do not originate from the brain and instead reflect an effect of psychoplastogens on BDNF in megakaryocytes, from which platelets appear to inherit most of their stored BDNF at the beginning of their 7–10 day lifespan [27]. Overall, though we found no evidence of a rapid increase in BDNF, previous research suggests that slow increases in peripheral BDNF may be specifically associated with successful antidepressant treatment.

Other moderators showing significant effects suggested that reported effects of psychoplastogens on BDNF were reduced with more rigorous study designs. In the analysis of all psychoplastogens, studies with more missing BDNF values tended to report bigger effect sizes. Additionally, studies calculating changes in BDNF using both a placebo control and change from baseline reported lower effect sizes, and studies with a high risk of bias rating showed bigger effect sizes than studies with a low risk of bias. For ketamine studies, those using randomized controlled designs reported lower effect sizes than non-randomized open-label trials. Taken together, these findings suggest that more rigorously controlled studies tend to find reduced effects of psychoplastogens on BDNF.

Preclinical research suggests that psychoplastogens can enhance BDNF levels and other aspects of neuroplasticity in the brain within a few hours of exposure [11]. The lack of an effect on peripheral BDNF in humans may thus seem unexpected. We propose two potential explanations. Firstly, it is possible that peripheral BDNF levels are not valid or reliable markers of short-term changes in brain BDNF and are thus unsuitable for measuring the purportedly rapid effects of psychoplastogens on neuroplasticity. Alternatively, it is possible that the pro-neuroplastic effects of psychoplastogens observed in animals may not occur in humans, or not to the same degree. Below we discuss both of these possibilities, as well as the limitations of this analysis and avenues for future research assessing rapid neuroplasticity changes after psychoplastogen intake in humans.

### Reliability and validity of peripheral BDNF as a marker of drug-induced neuroplasticity

There are several reasons to doubt peripheral BDNF as an informative biomarker in studies of psychoplastogen-induced neuroplasticity. Many commercially available BDNF assays, including some used in the studies we analyzed, show high inter-assay variability [35]. This is in addition to the questionable reliability of BDNF itself, which can vary strongly with sample handling (e.g. storage time, hemolysis), experimental parameters (e.g. time of day), and unavoidable fluctuations in participant characteristics (e.g. hormones, sleep deprivation, exercise, or recent smoking and drinking) [33, 34, 64]. Plasma BDNF also may be less reliable than serum BDNF, though it is questionable whether rapid changes in BDNF can be assessed in serum at all because most serum BDNF may be inherited from megakaryocytes [27, 65]. Studies can control for some of these factors, for example by instructing participants to avoid certain activities, including covariates in analyses, or excluding outlier values which might be due to hemolysis. However, it is difficult to account for all of these factors, and most studies used in this analysis did not describe how they handled outliers or managed known covariates of BDNF. One potential avenue for future research would be to more consistently distinguish between pro-BDNF and mBDNF, which may show different or even opposite associations with depressive symptoms and neuroplastic processes and may thus respond differently to psychoplastogens [66].

Furthermore, the validity of peripheral BDNF for reflecting brain BDNF is uncertain because it is not clear how much peripheral BDNF originates in the brain, and the proportion may be quite low. BDNF is a large molecule which does not diffuse easily across the blood-brain barrier, and the amount transported into the bloodstream may be very small [32]. Peripheral BDNF reflects secretions from other organs than the brain, and it is also mostly stored in platelets and thus affected by platelet count and activation [67]. Additionally, there is some evidence that serum BDNF mostly originates from megakaryocytes [27]. In rats and pigs, peripheral and hippocampal BDNF levels moderately correlate with each other [68]. However, several studies have failed to find overall correlations between central (i.e., cerebrospinal fluid) and peripheral BDNF in humans [69, 70]. In rodents, ketamine is also able to rapidly increase plasma BDNF levels without a corresponding increase in brain BDNF [71]. Peripheral BDNF may not be sensitive enough to reflect changes in BDNF expression in brain tissue at all, and platelets contain 100-1000-fold more BDNF than brain tissue [27, 72]. Thus, it may be that peripheral BDNF levels do not allow much inference about brain BDNF in the first place.

Another possibility is that peripheral BDNF levels correlate with brain BDNF under normal circumstances but do not reflect rapid changes in plasticity, such as those induced by psychoplastogens in animals. Rather, they may reflect longer-term changes in plasticity and BDNF homeostasis [73]. Psychoplastogens have rapid antidepressant effects and are thought to rapidly (<24 h) stimulate neuroplasticity, including BDNF expression [7]. However, if this is the case in the human brain, it may not be immediately reflected in peripheral BDNF. Overall, it is possible that peripheral BDNF does not reflect brain BDNF very well in humans, or that it is at least unsuitable for assessing rapid changes in brain BDNF.

### The “rat park” problem of drug-induced neuroplasticity

Another potential explanation for our results is that findings from animal research on psychoplastogens may not translate well to humans at all, at least not under all circumstances. Neurophysiological differences between species are always a possible explanation for differences between preclinical and human studies, though it is notable that BDNF structure and function are highly conserved across vertebrates [74]. Another possible confounder is laboratory conditions, specifically the stark differences in environment between laboratory animals and human study participants. To the best of our knowledge, no preclinical studies have reporting examining psychoplastogen-induced neuroplasticity under anything other than standard conditions [7, 9, 15]. Standard laboratory conditions for animal studies consist of relatively small cages with bedding and access to food and water, and animals may be housed alone or with a few same-sex animals. However, this unstimulating environment may negatively impact neuroplasticity compared to an animal’s natural setting, as seen in studies of enriched environments. Enriched environments, originally developed in from the “rat park” studies of drug dependence, are larger than standard cages and provide novel objects and toys, exercise equipment, and/or low-stress social interaction [75]. Compared to animals living in standard conditions, those living in enriched environments show increased neuroplasticity, including enhanced BDNF signaling, increased synapse size and neurogenesis, and increased dendritic branching, length, and spine numbers [75–77]. Living environment could greatly impact the translational validity of neuroplasticity studies given the stark contrast between a standard laboratory animal environment and an average human environment, which might be duly described as “enriched.” Neuroplasticity-enhancing drugs may encounter a ceiling effect, after which further enhancement is hardly possible [6]. If animals living in standard conditions have reduced neuroplasticity to begin with, they could be more responsive to drug-induced neuroplasticity than wild animals of the same species, or indeed, human beings.

### How can we measure psychoplastogen-induced neuroplasticity in humans?

If BDNF may not be a reliable and valid marker of drug-induced neuroplasticity, other methods involving neuroimaging and electrophysiology might be better tools for examining potential effects of psychoplastogens in humans. These methods also have the advantage of being more specific to brain regions and neuroplastic processes than a general peripheral marker like BDNF.

Structural imaging with magnetic resonance imaging or positron emission tomography can assess structural neuroplastic changes after a drug intervention, though these may not always reflect enhanced capacity for neuroplasticity per se. Synaptic growth can also be assessed using positron emission tomography by targeting synaptic vesicle glycoprotein 2 A (SV2A) [78]. A few studies have already examined the effects of psychoplastogens on structural neuroplastic changes. Ketamine may cause neuronal growth in some brain regions of both healthy subjects and patients, particularly the hippocampus [79–82]. Like with BDNF, however, some studies also find no change or even volume decreases [83–85]. A recent systematic review suggested that ketamine’s antidepressant effect may be associated with functional neuroplastic changes in the prefrontal cortex, frontoparietal regions, and striatum, which warrant further investigation [86]. Additionally, a study assessing SV2A density after ketamine treatment found no change in any healthy or patient group overall, though patients who began with lower SV2A density showed a significant increase in SV2A density in the dorsolateral prefrontal cortex and anterior cingulate cortex [87]. This would support the hypothesis that drug-induced neuroplasticity is most visible when a deficit is present at baseline.

Neuroplastic changes in functional connectivity can similarly be assessed using functional magnetic resonance imaging or magnetoencephalography, as well as electroencephalography [88, 89]. Lasting changes in functional connectivity have been reported after treatment with psilocybin and ayahuasca in healthy subjects [90–92]. Additionally, ketamine appears to post-acutely increase functional connectivity in regions important for emotional regulation and reward processing in depressed patients, although findings are somewhat mixed and the literature is small [93].

Finally, stimulation-based methods involving electrophysiology, such as paired associative stimulation or sensory-induced long-term potentiation (LTP), can be used to assess neuroplasticity in humans [94, 95]. These methods induce short-lived functional changes resembling LTP or long-term depression, the amplitude of which is an index of the capacity for neuroplasticity, and are able to measure the brain’s capacity for neuroplastic changes rather than simply the changes themselves. They are also sensitive to known correlates of neuroplasticity, including age, depression, physical activity, and short-term drug effects [96]. Sensory LTP in particular has been used in several studies of psychoplastogens, which have found increased visual LTP in depressed patients following ketamine treatment [97], as well as some evidence of complex neuroplastic effects in depressed patients receiving psilocybin [98] and healthy men microdosing LSD [99]. Overall, neuroimaging and electrophysiological methods likely offer more promising and specific avenues for exploring psychoplastogens’ effects on neuroplasticity than a peripheral marker like BDNF.

### Limitations

The results of this meta-analysis should be interpreted with certain limitations in mind. Firstly, handling and storage of BDNF samples can strongly impact results, and information on how this was controlled for was not always available. As discussed above, variations in the sensitivity and reliability of BDNF assays for mBDNF and its precursor proteins may introduce a great deal of noise, and most studies did not mention controlling for known covariates of BDNF. Additionally, one should consider the variation available in the potential moderator variables included. There was relatively little variation in dose, especially for ketamine. Studies of LSD and psilocybin in particular showed little variation in average participant age. Most studies also measured BDNF within a relatively short period of time after treatment (<48 h). This is the time frame in which preclinical studies observe rapid changes in brain BDNF [11]. However, a previous meta-analysis of ketamine treatments in depressed patients suggested that changes in peripheral BDNF in humans may only be visible on a larger time scale, e.g. several weeks, similar to BDNF changes following slower-acting antidepressant treatments. Relatedly, we did not have many studies with multiple ketamine infusions, and the ones we were able to include had a small (<7) number of infusions. Furthermore, this meta-analysis did not differentiate between treatment responders and non-responders in the studies of patients. It is possible that the effects of psychoplastogens on BDNF unfold over a longer timescale than analyzed here, and that they may be relatively specific to treatment responders who had been depressed. Similarly, psychoplastogens may act differently on different patient populations, but some patient groups (anxiety disorders, OCD) were too small to be analyzed here. Other moderator analyses that could not be conducted due to a lack of available data include the impact of smoking, medical comorbidity, and concurrent medication.

Additionally, we chose to analyze the effects of all known psychoplastogens together, given findings suggesting that they share downstream mechanisms of action involving glutamatergic signaling and remodeling of the extracellular matrix [20, 100, 101]. This is a similar approach to other meta-analyses analyzing multiple treatments expected to have similar effects on BDNF, for example antidepressant therapies [46–48]. However, it is possible that some psychoplastogens are more effective than others. We were only able to analyze ketamine, LSD, and psilocybin as separate drugs, and the number of studies was still relatively small for LSD and psilocybin.

## Conclusion and outlook

Taken together, this analysis demonstrates that the rapid increase in neuroplasticity after psychoplastogens observed in preclinical studies was not found in human studies which used peripheral BDNF as a biomarker. Psychoplastogens as a whole, but also the subgroup of classic psychedelics and the individual substances ketamine, LSD, and psilocybin all showed no significant effect on peripheral BDNF. It is possible that peripheral BDNF is an unsuitable biomarker for rapid, psychoplastogen-induced neuroplasticity. Additionally, an increase in neuroplasticity may be most visible when neuroplastic deficits are present, which could cause some problems with translating findings from animals in standard laboratory environments to humans. These findings are important because peripheral BDNF is widely used as a marker of rapidly enhanced neuroplasticity in studies of psychoplastogens, and its usefulness in this regard appears extremely limited. If we are to determine whether psychoplastogens rapidly enhance neuroplasticity in humans like they appear to do in animals, neuroimaging and stimulation-based methods may be more informative than peripheral markers like BDNF. As it stands, peripheral BDNF gives us no discernible evidence of increased neuroplasticity in humans after psychedelics and other psychoplastogens.

## Supplementary information

Supplementary information

## Data Availability

The datasets analysed during the current study are available in the Open Science Framework (OSF) repository, https://osf.io/t7d4c/.

## Code availability

R code used for this analysis is available in the Open Science Framework (OSF) repository, https://osf.io/t7d4c/.
