# Supplementary material for: Effects of psychoplastogens on blood levels of brain-derived neurotrophic factor (BDNF) in humans: a systematic review and meta-analysis
Source: Mol Psychiatry. 2024 Nov 29;30(2):763–76. doi: 10.1038/s41380-024-02830-z (PMC11753367; doi:10.1038/s41380-024-02830-z)
Supplement: Supplementary file 1 — Supplementary information [file 41380_2024_2830_MOESM1_ESM.pdf]

Supplementary Information

Supplement to: Calder A, Hase A & Hasler G. (2024) Effects of Psychoplastogens on Blood Levels of Brain-Derived Neurotrophic Factor (BDNF) in Humans: A Systematic Review and Meta-Analysis.

ID	Authors	Overall	D1	D2	D3	D4	D5	DS
Crossover Design								
1	Becker et al. (2022a)	+	+	+	+	+	!	+
2	Becker et al. (2022b)	+	+	+	+	+	!	+
3	Caliman-Fontes et al. (2023)	+	+	+	+	+	!	+
4	Glue et al. (2020b)	+	+	+	+	+	!	+
5	Holze et al. (2020)	+	+	+	+	+	!	+
6	Holze et al. (2021)	+	+	+	+	+	!	+
7	Holze et al. (2022)	+	+	+	+	+	!	+
8	Hutten et al. (2021)	-	+	+	-	+	!	+
9	Ley et al. (2023)	+	+	+	+	+	!	+
10	Park et al. (2019)	+	+	+	+	+	!	+
11	Straumann et al. (2023)	+	+	+	+	+	!	+
12	Vogt et al. (2023)	+	+	+	+	+	!	+

+ Low risk
! Some concerns
- High risk

D1 Randomisation process
DS Bias arising from period and carryover effects
D2 Deviations from the intended interventions
D3 Missing outcome data
D4 Measurement of the outcome
D5 Selection of the reported result

ID	Authors	Overall	D1	D2	D3	D4	D5
Parallel Design							
13	Allen et al. (2015)	!	!	!	+	+	!
14	de Almeida et al. (2019)	-	!	-	-	+	-
15	Glue et al. (2020a)	+	+	!	+	+	!
16	Grunebaum et al. (2017)	+	+	!	+	+	+
17	Grunebaum et al. (2018)	+	+	+	+	+	!
18	Haile et al. (2014)	!	+	+	!	+	!
19	Medeiros et al. (2021)	+	+	+	+	+	!
20	Rocha et al. (2021)	-	+	+	-	+	!
21	Woelfer et al. (2020)	!	+	+	!	+	!

+ Low risk
! Some concerns
- High risk

D1 Randomisation process
D2 Deviations from the intended interventions
D3 Missing outcome data
D4 Measurement of the outcome
D5 Selection of the reported result

ID	Authors	Overall	D1	D2	D3	D4	D5	D6	D7
Nonrandomized Design									
22	Duncan et al. (2013)	-	-	+	+	+	+	+	-
23	Glue et al. (2020c)	-	-	+	+	+	+	+	!
24	Kang & Vasquez (2021)	-	-	+	+	+	!	+	!
25	Linkovski et al. (2018)	-	-	+	+	+	!	+	!
26	Machado-Vieira et al. (2009)	-	-	+	+	+	+	+	!
27	Rybakowski et al. (2013)	-	-	+	+	+	+	+	!
28	Wang et al. (2021)	-	-	+	+	+	!	+	-
29	Zheng et al. (2021)	-	-	+	+	+	!	+	!

+ Low risk
! Some concerns
- High risk

D1 Bias due to confounding
D2 Bias in selection of participants into the study
D3 Bias in classification of interventions
D4 Bias due to deviations from intended interventions
D5 Bias due to missing data
D6 Bias in measurement of outcomes
D7 Bias in selection of the reported result

Figure S1. Results of the risk of bias assessment.

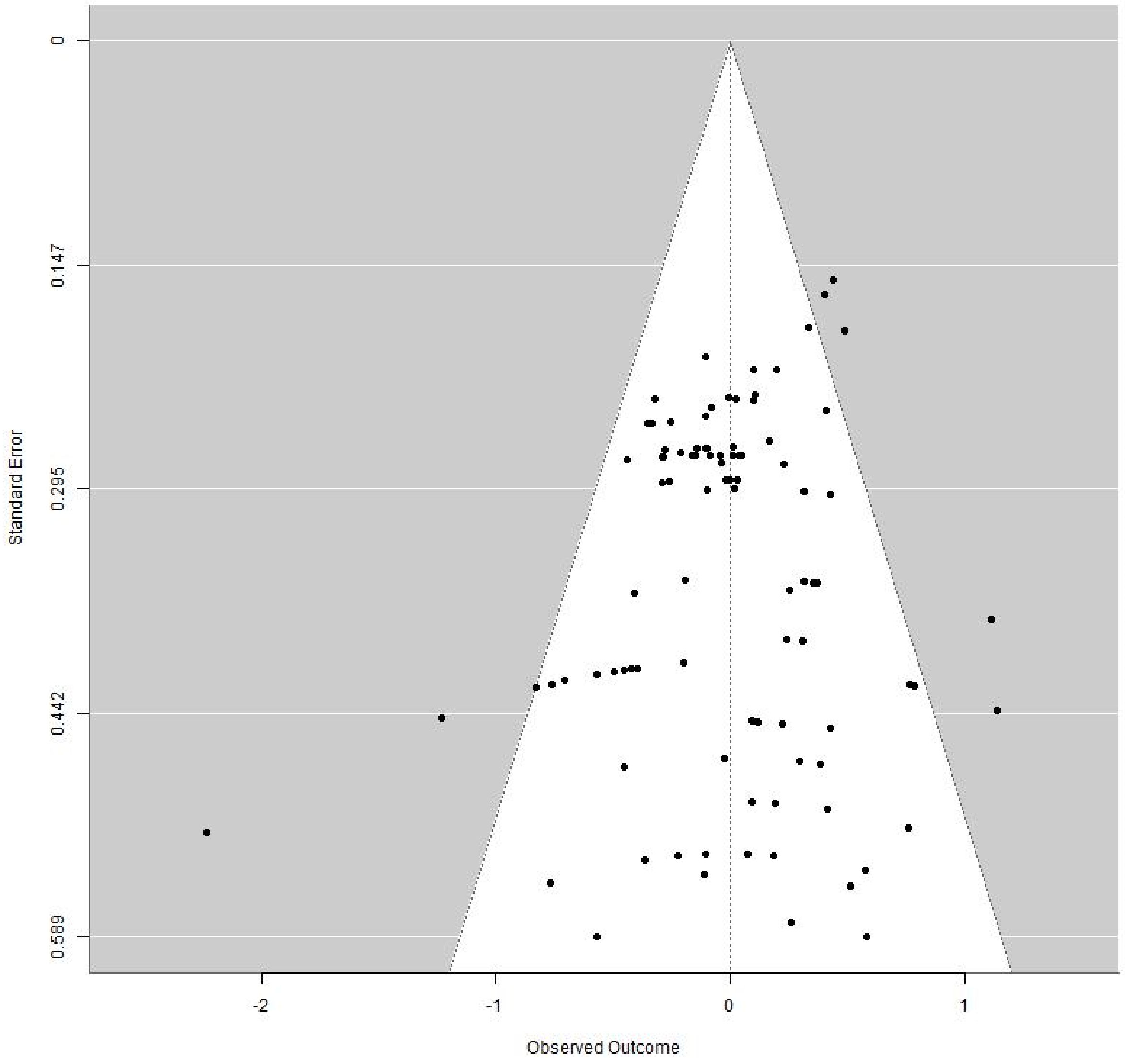

Figure S2. Funnel plot showing effect sizes and standard errors included in the meta-analysis of all psychoplastogens. Egger's test indicated no evidence of publication bias (p = 0.15).

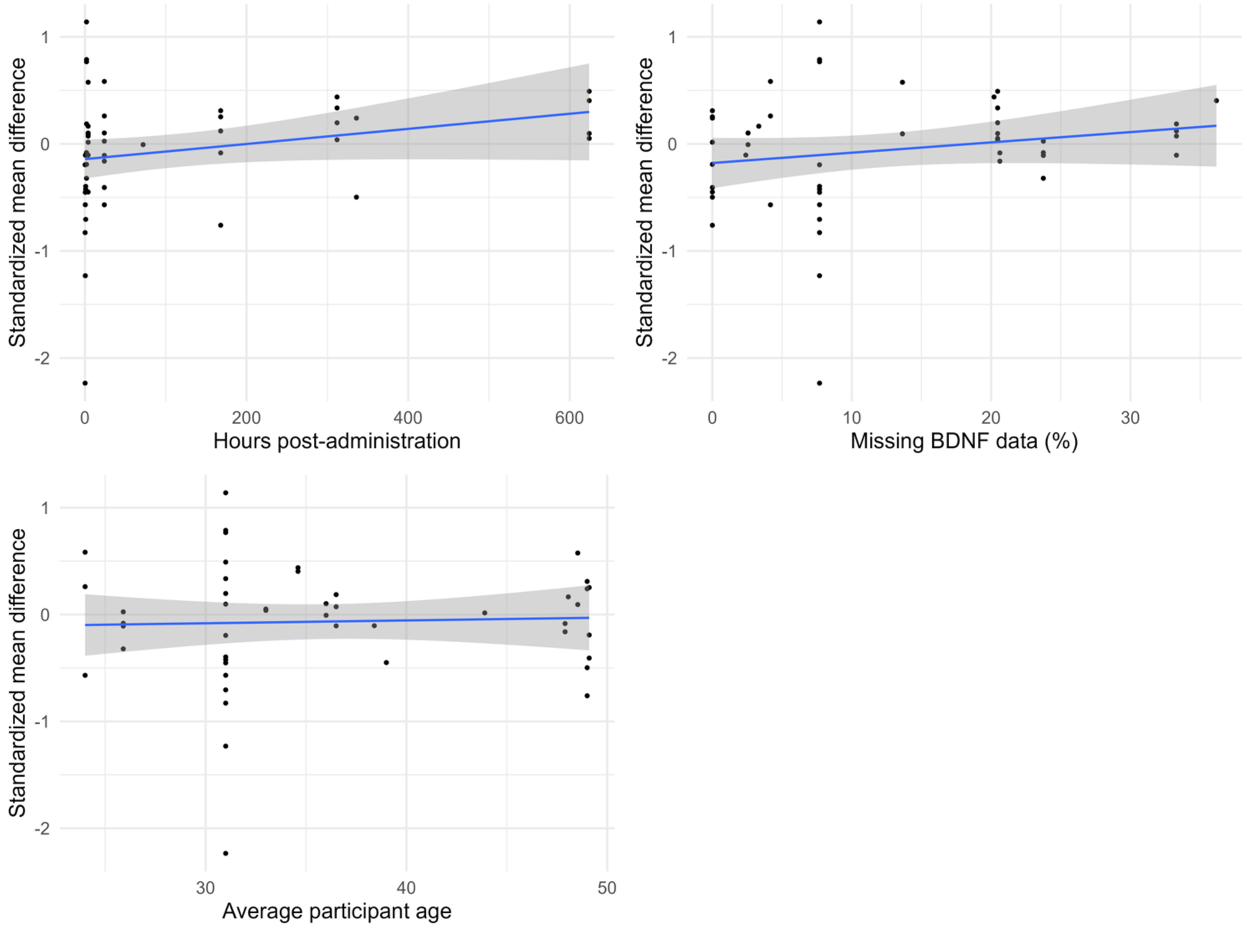

Figure S3. Relationship between effect sizes and the continuous variables of timing, percentage of missing values, and average participant age for studies administering ketamine. Shading shows 95% confidence intervals. The effect of hours post-administration was significant but extremely small (SMD = 0.0007, p = 0.015).

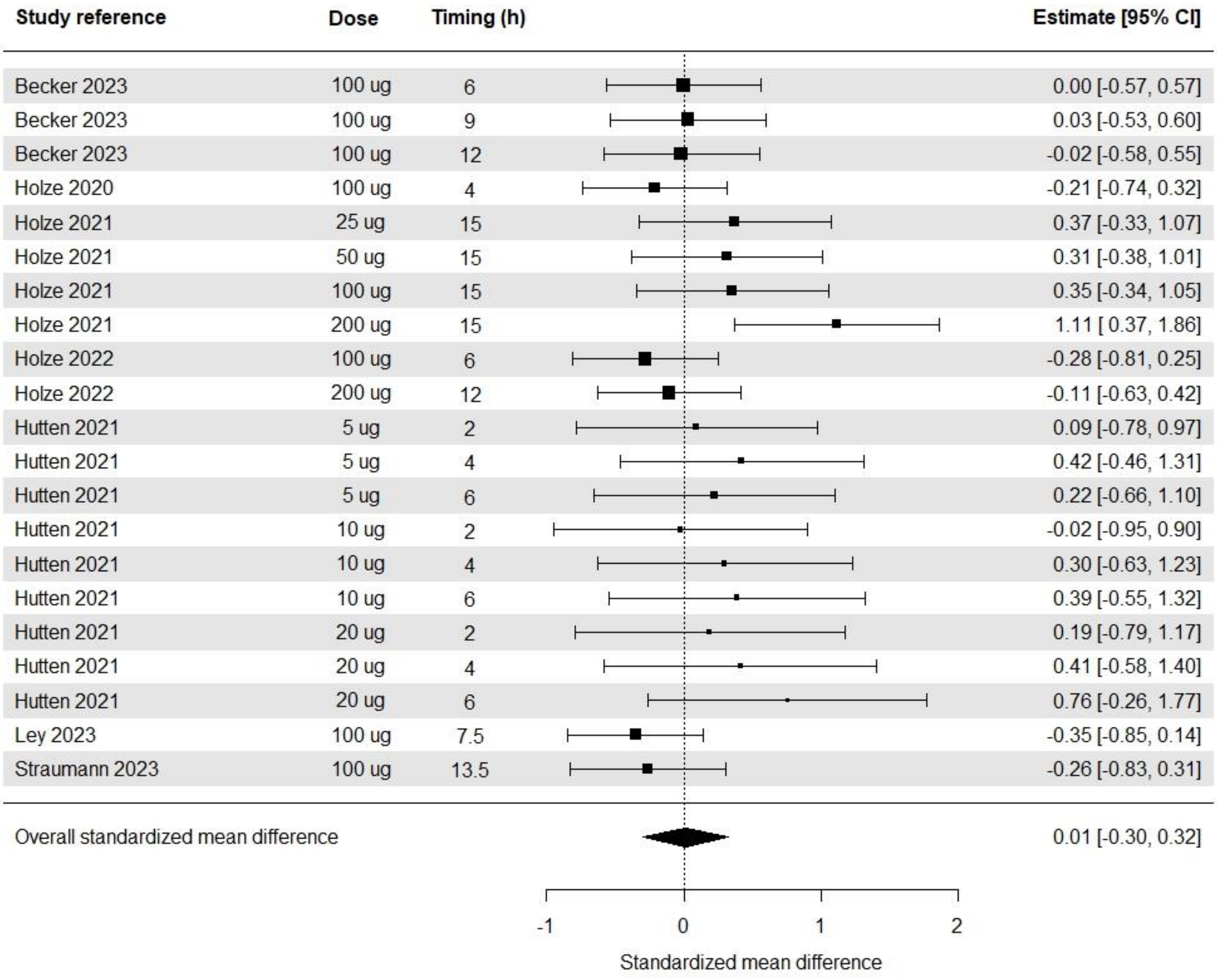

Figure S4. Forest plot showing overall effect size estimates of the change in BDNF after administration of LSD.

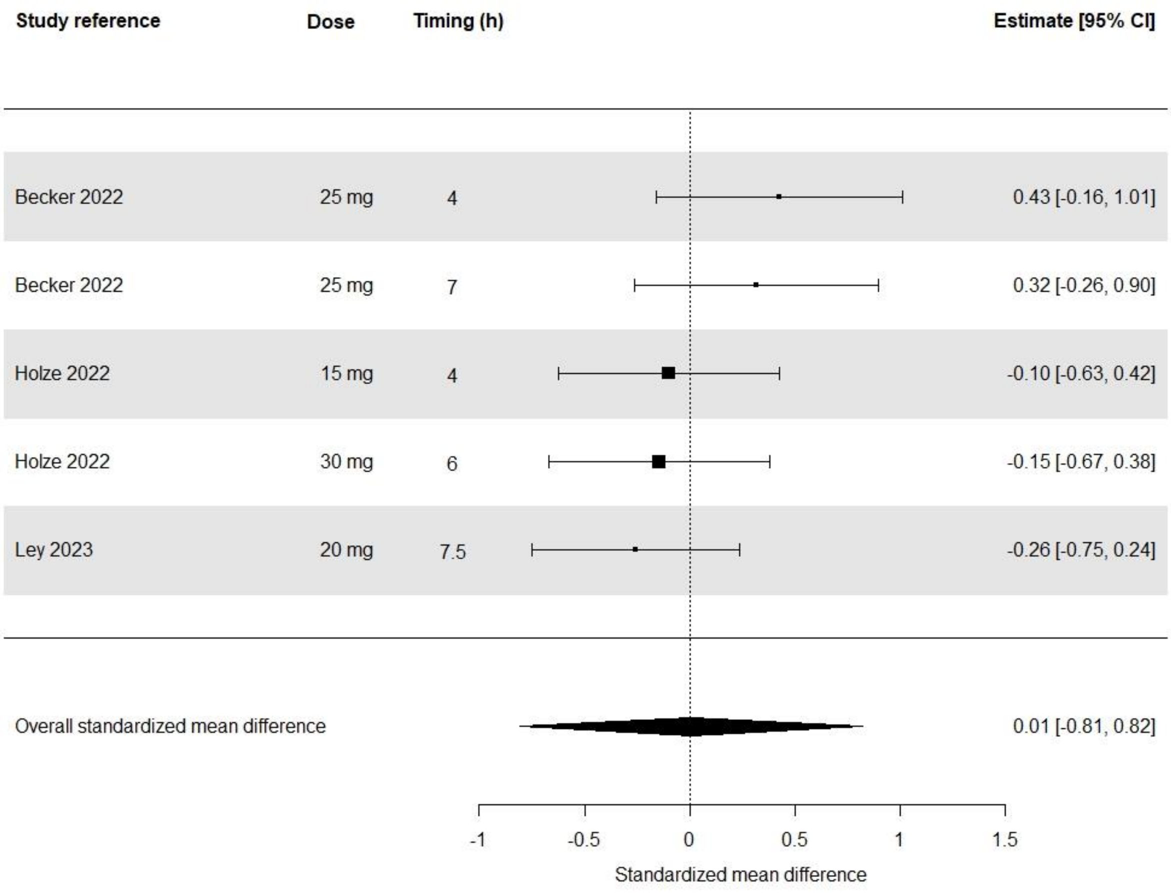

Figure S5. Forest plot showing overall effect size estimates of the change in BDNF after administration of psilocybin.

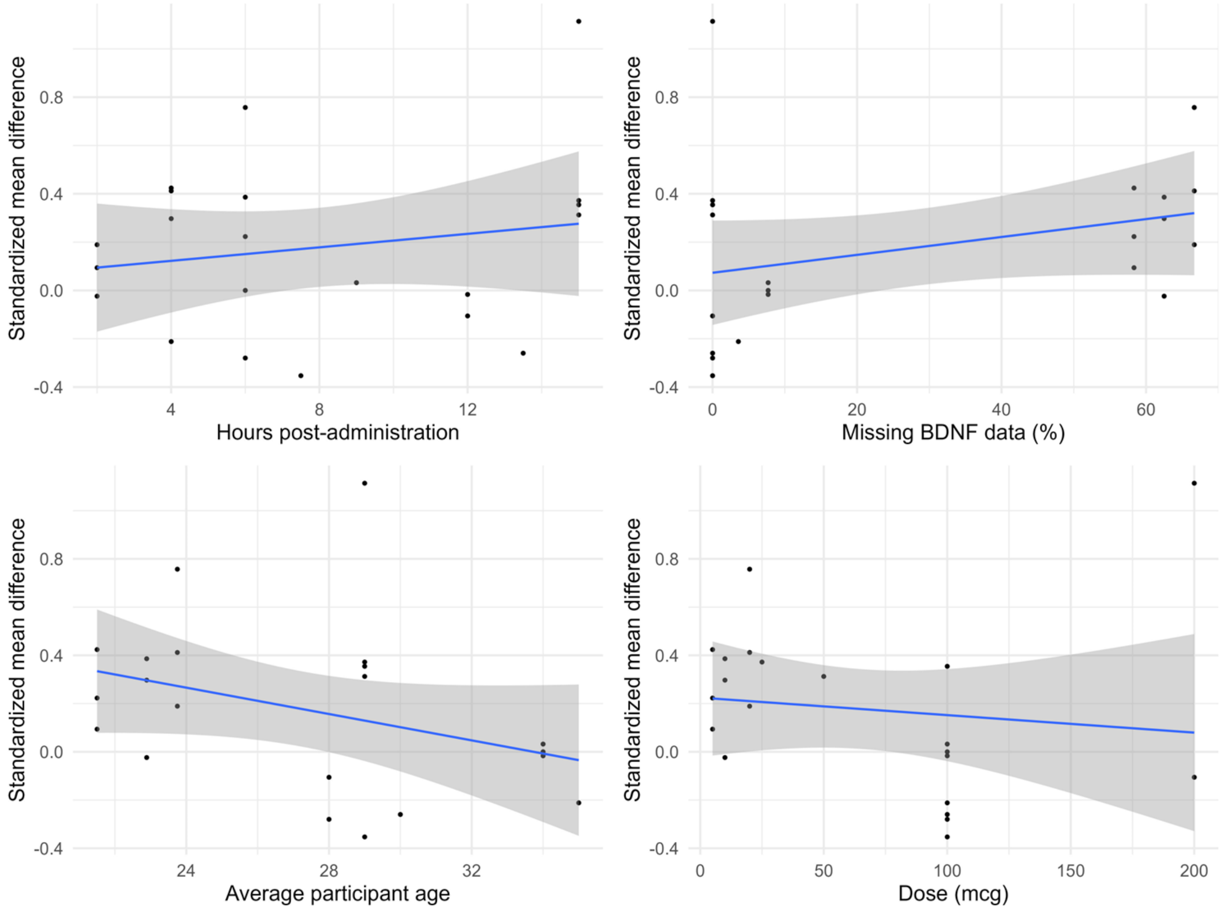

Figure S6. Relationship between effect sizes and the continuous variables of timing, percentage of missing values, average participant age, and dose for studies administering LSD. Shading shows 95% confidence intervals. No moderators showed a significant impact on effect sizes.

Supplementary information on BDNF Immunoassays

Table S1: Available information on sensitivity to mBDNF and proBDNF for immunoassays used in this meta-analysis. Information was taken from manufacturers' websites (accessed 15.07.2024) and from an independent validation study.

**Manufacturer**	**Immunoassay**	**Sensitivity**
Biosensis	Mature BDNF Rapid ELISA kit (no.BEK-2211-2P)	mBDNF with minimal cross-reactivity to proBDNF (Manufacturer's website)
Chemicon International	anti-BDNF sandwich ELISA kit	Not specified
Millipore	ChemiKine Sandwich ELISA (no. CYT306)	Total BDNF[1] (Manufacturer's website)
MesoScale Discovery	custom assay	Not specified
Promega	BDNF ELISA kit (no. G7610)	Total BDNF[1] (Manufacturer's website)
R&D Systems	DuoSet ELISA Development Kit (no. DY248)	Total BDNF (Manufacturer's website)
R&D Systems	Total BDNF Quantikine ELISA Kit (no. DBNT00)	Total BDNF (Manufacturer's website)

Supplementary references

1. Polacchini, A., Metelli, G., Francavilla, R., Baj, G., Florean, M., Mascaretti, L. G., & Tongiorgi, E. (2015). A method for reproducible measurements of serum BDNF: comparison of the performance of six commercial assays. *Scientific reports*, *5*(1), 17989.
